# Involvement of Parvalbumin-Positive Neurons in the Development of Hyperalgesia in a Mouse Model of Fibromyalgia

**DOI:** 10.3389/fpain.2021.627860

**Published:** 2021-02-26

**Authors:** Kenichiro Miyahara, Hiroshi Nishimaru, Jumpei Matsumoto, Tsuyoshi Setogawa, Toru Taguchi, Taketoshi Ono, Hisao Nishijo

**Affiliations:** ^1^System Emotional Science, Faculty of Medicine, University of Toyama, Toyama, Japan; ^2^Department of Physical Therapy, Faculty of Rehabilitation, Niigata University of Health and Welfare, Niigata, Japan; ^3^Institute for Human Movement and Medical Sciences, Niigata University of Health and Welfare, Niigata, Japan

**Keywords:** fibromyalgia, mice, pain sensitivity, parvalbumin-positive neurons, reserpine, somatosensory cortex

## Abstract

Fibromyalgia (FM) presents as chronic systemic pain, which might be ascribed to central sensitization, in which pain information processing is amplified in the central nervous system. Since patients with FM display elevated gamma oscillations in the pain matrix and parvalbumin (PV)-positive neurons play a critical role in induction of gamma oscillations, we hypothesized that changes in PV-positive neurons are involved in hyperalgesia in fibromyalgia. In the present study, to investigate a role of PV-positive neurons in neuropathic pain, mice received reserpine administration for 3 consecutive days as an animal model of FM (RES group), while control mice received vehicle injections in the same way (VEH group). The mice were subjected to hot-plate and forced swim tests, and immuno-stained PV-positive neurons were counted in the pain matrix. We investigated relationships between PV-positive neuron density in the pain matrix and pain avoidance behaviors. The results indicated that the mice in the RES group showed transient bodyweight loss and longer immobility time in the forced swim test than the mice in the VEH group. In the hot-plate test, the RES group showed shorter response latencies and a larger number of jumps in response to nociceptive thermal stimulus than the VEH group. Histological examination indicated an increase in the density of PV-positive neurons in the primary somatosensory cortex (S1) in the RES group. Furthermore, response latencies to the hot-plate were significantly and negatively correlated with the density of PV-positive neurons in the S1. These results suggest a critical role for PV-positive neurons in the S1 to develop hyperalgesia in FM.

## Introduction

Fibromyalgia (FM) presents with chronic systemic pain along with psychotic (e.g., depression) and autonomic nervous symptoms (1–4). Epidemiological studies of FM in various countries have reported an average prevalence of 2.7% (5–8). However, FM is refractory, and its pathophysiological mechanisms are not fully understood. Treatment methods of FM are under development, and various pharmacological therapies combined with non-pharmacological therapies have been used (9–12). Recently, central sensitization, in which pain information processing is amplified in the central nervous system, has been suggested to play an essential role in FM (13–15).

Consistent with the above hypothesis, functional magnetic resonance imaging (fMRI) studies have reported hyperactivity in multiple brain areas that process pain, including the somatosensory area, prefrontal cortex, anterior cingulate cortex, and insula in response to mechanical, thermal and electrical stimulation in patients with FM as well as an animal model of FM (16–19). Neurophysiological studies also reported that excitability in the primary somatosensory (S1) cortex was increased in patients with FM (20, 21). Furthermore, gamma oscillations in S1 were correlated with subjective pain (or behavioral responses to nociceptive stimuli in rats) and/or physical stimulus intensity in intact humans and rats (22–27). Gamma oscillations were elevated in the S1, motor cortex, insula, and prefrontal cortex in patients with FM compared with controls (28).

Several animal models of FM have been reported. Repeated injection of reserpine, which results in the depletion of monoamines in the nervous system, has been used as an animal model of FM (29, 30). In this model, the animals showed behaviors associated with pain (hyperalgesia and allodynia), depression-like symptoms, and gastrointestinal dysfunction (autonomic symptoms), which are all observed in human FM. Furthermore, reserpine administration increased the responses of mechanoreceptive C-nociceptors and the activity of dorsal horn microglia in the spinal cord (31). These previous human and animal studies suggest that the forebrain pain matrix might be hyperactive to display complex FM symptoms. On the other hand, a recent animal study reported that optogenetic activation of parvalbumin (PV)-positive neurons in the S1 induced gamma oscillations of local field potentials and pain-related avoidance behaviors (32). Furthermore, optogenetic activation of PV-positive neurons in the prelimbic cortex also enhanced avoidance responses to nociceptive stimuli (33). Based on these findings, we hypothesized that PV-positive neurons play an essential role in pain information processing in FM. In this study, we investigated the relationship between PV-positive neurons in the forebrain pain matrix and pain sensitivity in an animal model of FM with repeated reserpine administration.

## Materials and Methods

### Subjects

Eight to 10-week-old C57BL/6J male mice (*n* = 60, Japan SLC, Hamamatsu, Japan) were used. The mice were housed in groups (four per cage) in a temperature-controlled experimental room (22 ± 1°C) with light control (lights on from 07:00 to 19:00) and food and water available *ad libitum*. The mice were treated consistently with the guidelines for care and use of laboratory animals approved by the University of Toyama and the National Institutes of Health's Guide for the Care and Use of Laboratory Animals. The experimental protocol of the study was approved by the Animal Experiments and Ethics Committee at the University of Toyama (Permit No. A2016MED-2 3).

### Animal Model of FM by Reserpine

An animal model of FM was produced using the protocol described in previous studies (31, 34, 35). Reserpine (Nacalai Tesque, Inc., Kyoto, Japan), adjusted to a concentration of 0.25 mg/mL with 0.5% acetic acid, was injected (0.25 mg/kg, s.c.) into the back skin once a day for 3 successive days (RES group). As a control, a vehicle solution (0.5% acetic acid) was similarly injected (VEH group).

### Behavioral Tests

#### Hot-Plate Test

Previous studies reported that gene expression of the acid-sensing ion channel 3 (ASIC3) was increased in the dorsal root ganglion of the same animal model of FM, and that a selective blocker of ASIC3 (APETx2) decreased both mechanical and thermal hyperalgesia (31, 36). Clinical studies reported that not only mechanical but also thermal hyperalgesia are important factors predicting clinical pain intensity in patients with chronic pain including FM (37, 38). In the present study, thermal hyperalgesia (avoidance latency) was assessed using the hot-plate test, data of which were directly applied to correlational analyses with PV-positive neuron density (see below).

Previous studies reported that pain hypersensitivity (mechanical allodynia) was detected 3 days after the first reserpine injection in this animal model and gradually returned to the baseline levels on 10th to 14th day after the first reserpine injection (29, 31). Therefore, behavioral responses to noxious thermal stimuli were observed 3 days after the first injection in the present study. After placing each mouse on a hot-plate apparatus (Muromachi Kikai, Japan), the latency of behavioral responses [hindpaw licking or jumping (whichever came first)], and the number of jumps were measured. The surface temperature of the hot-plate was set at 50 ± 0.5°C before testing, and the test was completed in 60 s to avoid tissue damage to the animals.

#### Forced Swim Test

Previous studies reported that depression-like behaviors in the forced swim test were not observed 3 days after the first reserpine injection, but observed 5–14 days after the first reserpine injection (29, 34, 35). In the present study, two different groups of mice underwent the forced swim test 3–4 and 10−11 days after the first injection to determine the depressive behaviors caused by reserpine. The procedures were conducted in accordance with those in Porsolt et al. (39). Each mouse was placed in water (25 ± 1°C) in a glass beaker (23 × 35 × 20 cm; diameter × height × depth) for 15 min 3 or 10 days after the first injection. Twenty-four hours after the first forced swim test (i.e., 4 or 11 days after the first injection), the mice were again placed in the same glass beaker with water for 5 min, and their behavior was recorded by a video camera. The immobility time was measured for 5 min in the second forced swim test. Immobility was defined as the absence of any movement except that to keep the mouse's head above the water. After testing, the animals were towel-dried and returned to their cages.

### Immunohistochemistry

PV-positive neurons were immunostained based on the same protocol used in our previous studies (40–44). After the hot-plate test was performed 3 days after the first injection, the mice were sacrificed under deep anesthesia with mixed anesthetics (5.0 mg/kg butorphanol, 4.0 mg/kg midazolam, and 0.75 mg/kg medetomidine, i.p.), by transcardial perfusion with heparinized 0.01 M phosphate buffer saline (PBS), followed by 4% paraformaldehyde dissolved in 0.1 M phosphate buffer (PB). After perfusion, the brains were post-fixed in 4% paraformaldehyde overnight. The fixed brain was then immersed in 30% sucrose until they sank to the bottom. Then, the brains were cut into 40 μm sections, collected in 0.01 M PBS, and stored in an antifreeze solution (25% glycerin, 25% ethylene glycol, and 50% 0.1 M PB) at −20°C. Two stains were used on serial sections every 40 μm, one for PV immunocytochemistry, and the other for Cresyl violet (Nissl staining). In PV immunostaining, the sections were processed with mouse monoclonal anti-PV antibodies according to our previous protocol (40–44). Briefly, the sections were washed 3 times with 0.01 PBS for 5 min, blocked with 3% normal horse serum for 30 min, then mouse monoclonal anti-parvalbumin antibody (1: 10 000 dilution in 1% horse serum PBS, Sigma, St. Louis, MO, USA) was incubated overnight at 4°C. These sections were washed 3 times with 0.01 PBS for 5 min and incubated with biotinylated horse anti-mouse IgG (1:200 dilution, Vector, Burlingame, USA) for 50 min at room temperature. After washing, incubated with avidin-biotin complex reagent (Vector) for 50 min and visualized with a detection solution (0.25 mg/ml 3, 3′-diaminobenzidine, 0.03% H_2_O_2_ in PB). Negative control sections were treated identically except for omission of the primary antibody. No reaction product was observed in any of the control sections.

### Stereological Analysis of PV-Positive Neurons

PV-positive neurons were counted based on our previous protocols (42–44). Section images were captured and digitized using a microscope system (BZ-9000, Keyence Corporation, Osaka, Japan). Anatomical locations of the brain areas were determined by examining the anatomically matched adjacent Nissl-stained sections based on the brain atlas (45); at +0.98, +0.62, +0.14, −0.34, −0.70, and −1.22 mm in the anterior-to-posterior level from the bregma in the primary somatosensory cortex (S1); at +1.98, +1.70, +1.42, +1.10 mm in the medial pre-frontal cortex (mPFC) including the prelimbic cortex (PrL), infralimbic cortex (IL) and anterior cingulate cortex (ACC); at −0.82, −1.22, −1.58, −1.94, −2.30 mm in the lateral (LA) and basolateral (BLA) nuclei of the amygdala; at−0.82, −1.58 mm in the intercalated cells of the amygdala (ITC); at +0.38, −0.22, −0.82, −1.22 mm in the granular insula (GI), dysgranular insula (DI), and agranular insula (AI). In S1, both the forelimb (S1FL) and hindlimb (S1HL) regions were separately analyzed.

The PV-positive neurons were counted using stereological software (Stereo Investigator version 7.53.1, MicroBrightField, Williston, VT, USA). The cell bodies of PV-positive neurons in the sample sites randomly dispersed in each brain region were counted using a 20× objective lens. The counting conditions were as follows; sampling grid sizes, 280.87 × 765.50-μm in the mPFC, and 259.00 × 372.40-μm in the S1, amygdala, and insula; counting frame, 200 × 200-μm; optical dissector height, 5 μm. The software automatically set up square counting frames with exclusion lines. Within the counting frame, only PV-positive cell bodies that did not contact the excluding line were counted. The detailed theoretical and technical methodology for stereological estimation of cell density has been previously reported (46). The PV-positive neuron density was estimated in each brain area of each animal.

### Statistical Analysis

Data were shown as the mean ± SEM. Normality of the data was checked by D'Agostino & Pearson test. The bodyweights were compared between the two groups using a repeated measures two-way analysis of variance (ANOVA) with *post-hoc* tests (Bonferroni tests). In this analysis, the degrees of freedom were corrected by Greenhouse–Geisser method where appropriate. Data in the behavioral tests and PV-positive neuron density were compared between the VEH and RES groups using unpaired *t*-tests with Welch's correction (Welch's test) except the data in numbers of jumps in the hot-plate test, immobility time in the forced swim test 11 days after the first injection, and PV-positive neuron density in the infralimbic cortex. The data in numbers of jumps in the hot-plate test, immobility time in the forced swim test 11 days after the first injection, and PV-positive neuron density in the infralimbic cortex were analyzed by the Mann–Whitney *U*-test because these data did not show normal distribution. A linear regression analysis was used to analyze the relationship between the response latencies in the hot-plate test and PV-positive neuron density. Prism 8 (GraphPad Software Inc.) was used to analyze the data. A *p* < 0.05 was considered statistically significant.

## Results

### Bodyweights of the Reserpinized Animals

**Figure 1:**
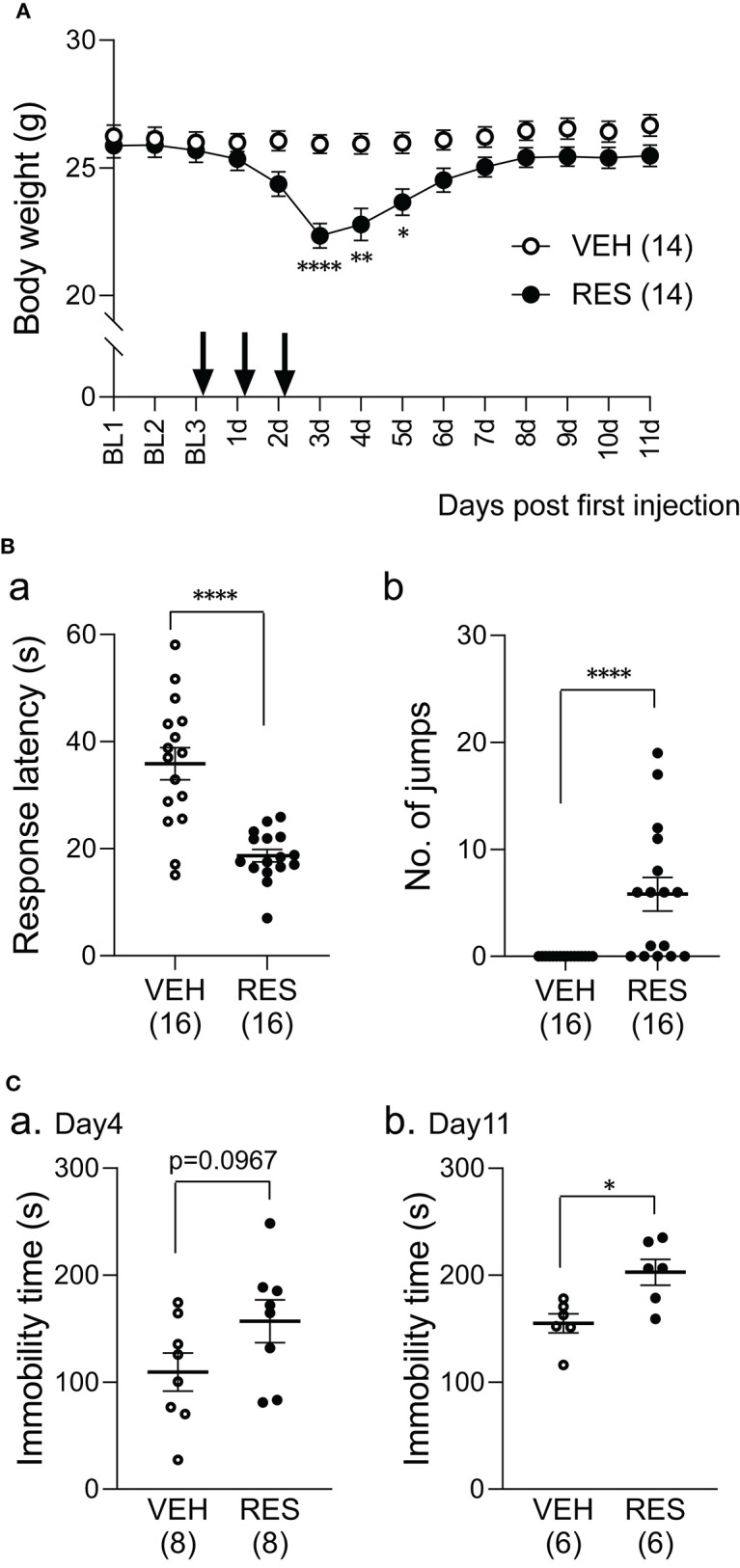
Effects of repeated reserpine injection on body weight **(A)** and behavioral responses **(B,C)**. **(A)** Time course of bodyweight. The mean bodyweights were significantly lower in the RES group than in the VEH group 3–5 days after the first injection (3, 4, and 5 d). ^****^, ^**^, ^*^, significant differences from the VEH group (Bonferroni test, *p* < 0.0001, 0.01, and 0.05, respectively). The arrows indicate reserpine or vehicle injection. Open circles, VEH group; filled circles, RES group. BL1-3, day 1–3 in the baseline period; 1–11 d, 1–11 days after the first injection. **(B)** Comparison of response latency (a) and the number of jumps (b) between the VEH and RES groups in response to the hot-plate test (a thermal stimulus) 3 days after the first injection. ^****^p < 0.0001 [(a) Welch's test; (b) Mann–Whitney *U*-test). Open circles, VEH group; filled circles, RES group. Numbers in parentheses indicate the number of animals. **(C)** Comparison of the immobility time between the VEH and RES groups in the forced swim test on day 4 (a) and day 11 (b) after the first injection. ^*^p < 0.05 (Mann–Whitney *U*-test). Open circles, VEH group; filled circles, RES group. Numbers in parentheses indicate the number of animals.

The bodyweight of the RES group decreased after reserpine injection (Figure 1A). The statistical analysis indicated significant main effects of group [*F*_(1, 26)_ = 5.54, *p* = 0.026] and day [*F*_(2.82, 73.27)_ = 49.22, *p* < 0.001], and a significant interaction between group and day [*F*_(13, 338)_ = 30.62, *p* < 0.0001]. *Post-hoc* comparisons indicated that the mean bodyweights were significantly smaller in the RES group than in the VEH group 3 days after the first injection (3 d in Figure 1; 22.3 ± 0.5 vs. 25.9 ± 0.4 g: mean ± SEM; Bonferroni test, *p* < 0.0001), 4 days after the first injection (4 d in Figure 1; 22.8 ± 0.6 vs. 25.9 ± 0.4 g; Bonferroni test, *p* = 0.0046), and 5 days after the first injection (5 d in Figure 1; 23.7 ± 0.5 vs. 26.0 ± 0.4 g; Bonferroni test, *p* = 0.0248).

### Behavioral Tests

#### Hot-Plate Test

The mice underwent the hot-plate test 3 days after the first injection (3 d in Figure 1A). The response latencies to nociceptive thermal stimuli were significantly shorter in the RES group (18.7 ± 1.2 s, *n* = 16) than in the VEH group (35.9 ± 3.0 s, *n* = 16) (Welch′s test, *p* < 0.0001; Figure 1Ba). The number of jumps was significantly greater in the RES group (5.8 ± 1.6 times, *n* =16) than in the VEH group (0.0 ± 0.0 times, *n* = 16; Mann–Whitney *U*-test, *p* < 0.0001; Figure 1Bb). These results indicate that pain sensitivity was increased in the RES group.

#### Forced Swim Test

Figure 1C shows the immobility time 4 and 11 days after the first injection (4 and 11 d in Figure 1A). On day 4, the immobility time in the RES group (156.9 ± 19.9 s, *n* = 8) tended to be longer than that in the VEH group (109.3 ± 17.7 s, *n* = 8; Welch′s test, *p* = 0.0967; Figure 1Ca). On day 11, the immobility time was significantly longer in the RES group (202.8 ± 12.1 s, *n* = 6) than in the VEH group (155.1 ± 8.9 s, *n* = 6; Mann–Whitney *U*-test, *p* = 0.0152; Figure 1Cb).

### PV-Positive Neuron Density

**Figure 2:**
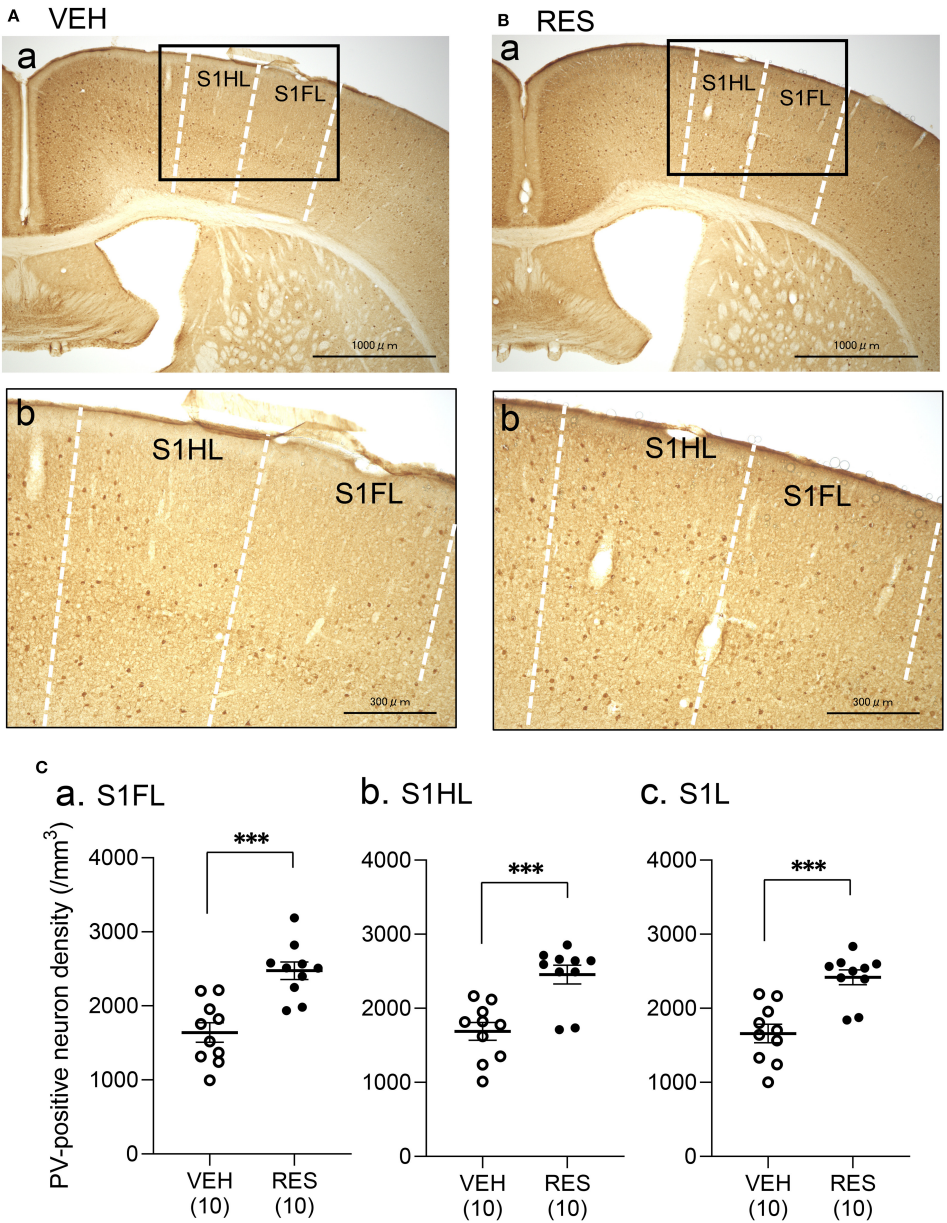
Effects of repeated reserpine injection on PV-positive neurons in S1. **(A,B)** Photomicrographs of the mice S1 in the VEH **(A)** and RES **(B)** groups. Insets in (a) are shown in (b) as enlarged views. The number of PV-positive neurons was increased in the RES group. S1HL, S1 hindlimb area; S1FL, S1 forelimb area. **(C)** Comparison of the PV-positive neuron density in the S1FL (a), S1HL (b), and S1L (c) between the VEH and RES groups. S1FL, S1 forelimb area; S1HL, S1 hindlimb area; S1L, S1 leg area (mean of S1FL and S1HL). ^***^p < 0.001 (Welch's test). Open circles, VEH group; filled circles, RES group. Numbers in parentheses indicate the number of animals.

**Figure 3:**
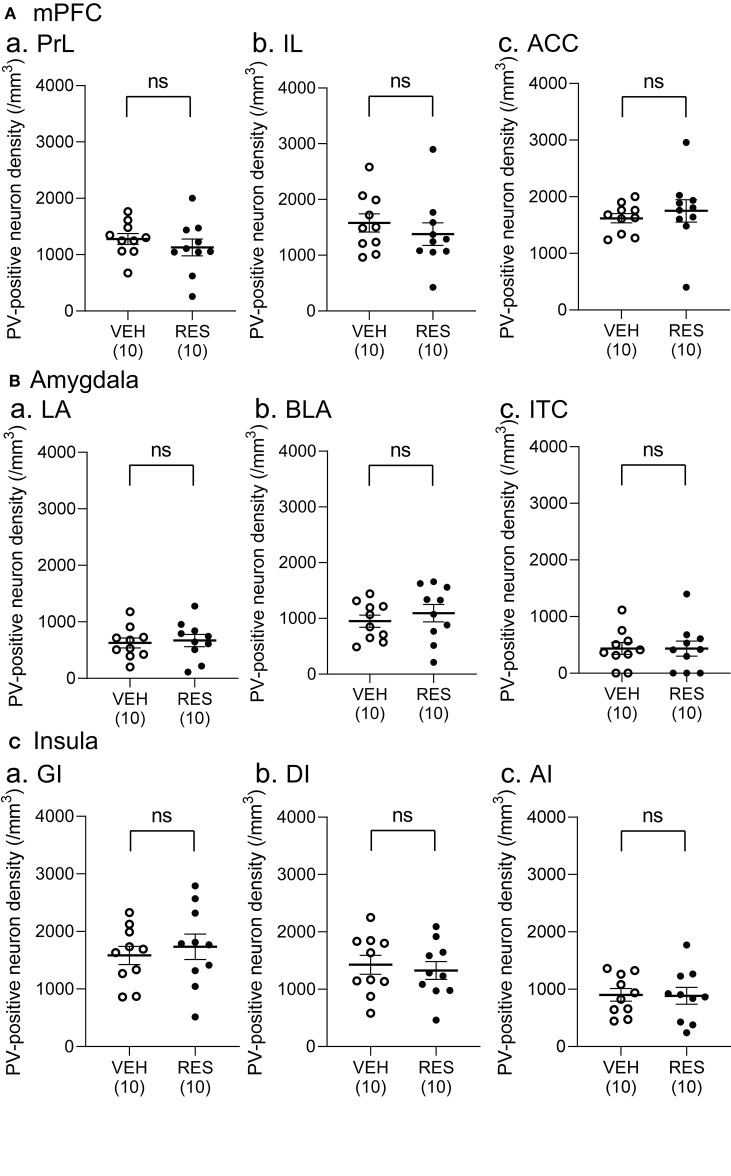
Comparison of the cell density of PV-positive neurons between the VEH and RES groups in the medial pre-frontal cortex (mPFC) **(A)**, amygdala **(B)**, and insula **(C)**. **(A)** PV-positive neuron density in the prelimbic cortex (PrL) (a), infralimbic cortex (IL) (b), and anterior cingulate cortex (ACC) (c) in the mPFC. **(B)** PV-positive neuron density in the lateral nucleus (LA) (a), basolateral nucleus (BLA) (b), and intercalated cells (ITC) (c) in the amygdala. **(C)** PV-positive neuron density in the granular insula (GI) (a), dysgranular insula (DI) (b), and agranular insula (AI) (c). There were no significant differences in the PV-positive neuron density between the VEH and RES groups in all regions (IL: Mann–Whitney *U*-test, *p* > 0.05; other brain regions: Welch's test, *p* > 0.05). Numbers in parentheses indicate the number of animals. ns, non-significant (*p* > 0.05).

Example microphotographs of PV-positive neurons in S1 for the VEH and RES groups are shown in Figures 2A,B. The number of PV-positive neurons was greater in the RES group than in the VEH group. Figure 2C shows PV-positive neuron density (cells/mm^3^) in the S1 forelimb (S1FL) (Figure 2Ca) and S1 hindlimb (S1HL) regions (Figure 2Cb), and cell density in the S1L (mean cell density between the S1FL and S1HL) (Figure 2Cc). The Welch′s test indicated that PV-positive neuron density was significantly greater in the RES group than in the VEH group in each area (S1FL, *p* = 0.0002; S1HL, *p* = 0.0004; S1L, *p* = 0.0002). However, in the other brain regions, no significant differences were observed. In the mPFC (Figure 3A), there were no significant differences in PV-positive neuron density between the RES and VEH groups in the prelimbic cortex (PrL) (Figure 3Aa), infralimbic cortex (IL) (Figure 3Ab), and anterior cingulate cortex (ACC) (Figure 3Ac) (IL: Mann–Whitney *U*-test, *p* > 0.05; other brain regions: Welch's test, *p* > 0.05). In the amygdala (Figure 3B), there were no significant differences between the RES and VEH groups in the lateral nucleus (LA) (Figure 3Ba), basolateral nucleus (BLA) (Figure 3Bb), and intercalated cells (ITC) (Figure 3Bc) (all regions: Welch's test, *p* > 0.05). In the insula cortex (Figure 3C), no significant differences were observed between the VEH and RES groups in the granular insula (GI) (Figure 3Ca), dysgranular insula (DI) (Figure 3Cb), and agranular insula (AI) (Figure 3Cc) (all regions: Welch's test, *p* > 0.05).

### Correlation Analyses

**Figure 4:**
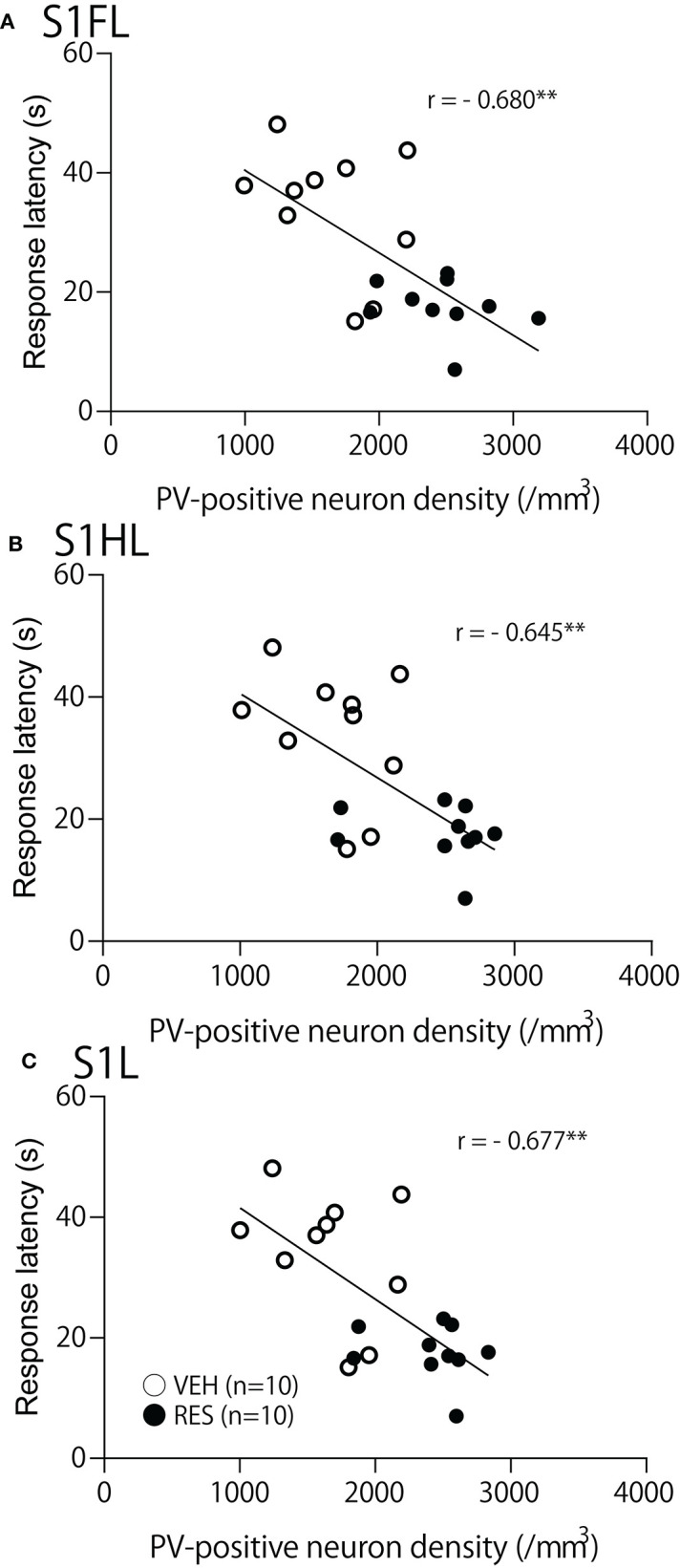
Relationships between response latency in the hot-plate test and PV-positive neuron density in the S1FL **(A)**, S1HL **(B)**, and S1L **(C)**. ^**^p < 0.01, respectively. Open circles, VEH group; filled circles, RES group. Other descriptions are shown in Figure 2.

**Figure 5:**
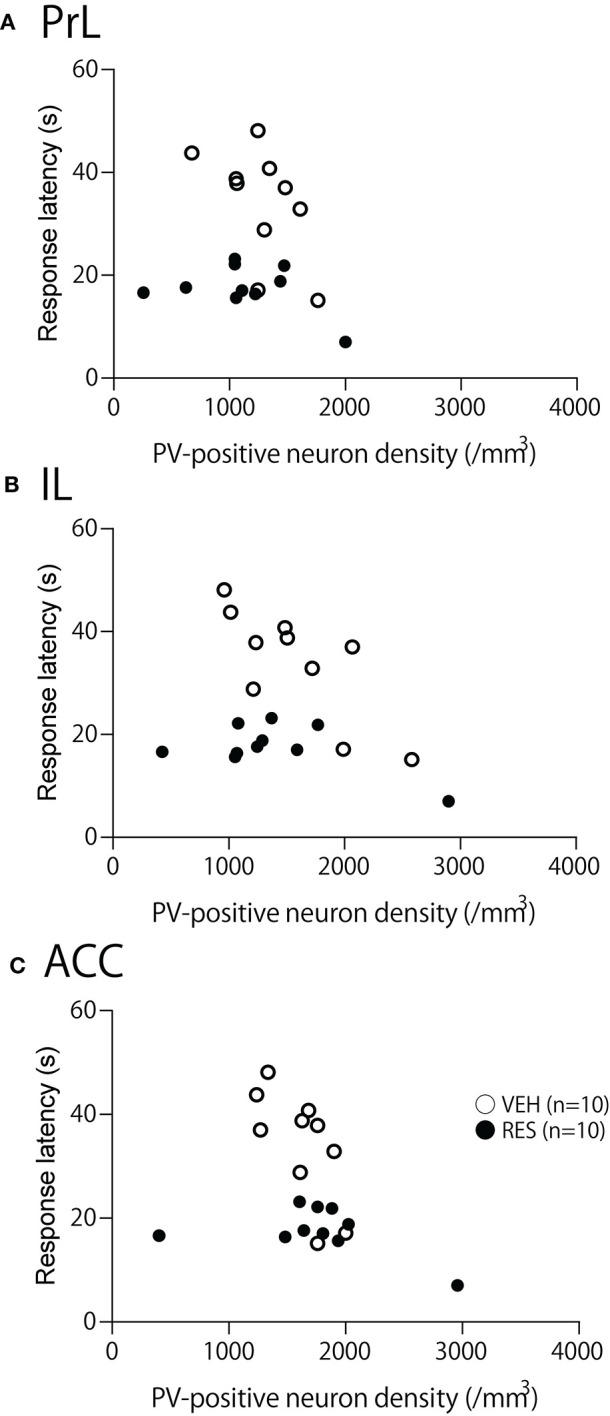
Relationship between the response latency in the hot-plate test and PV-positive neuron density in the prelimbic cortex (PrL) **(A)**, infralimbic cortex (IL) **(B)**, and anterior cingulate cortex (ACC) **(C)**. **(A–C)** There was no significant correlation between response latency and PV-positive neuron density in the PrL **(A)**, IL **(B)**, and ACC **(C)** (*p* > 0.05).

**Figure 6:**
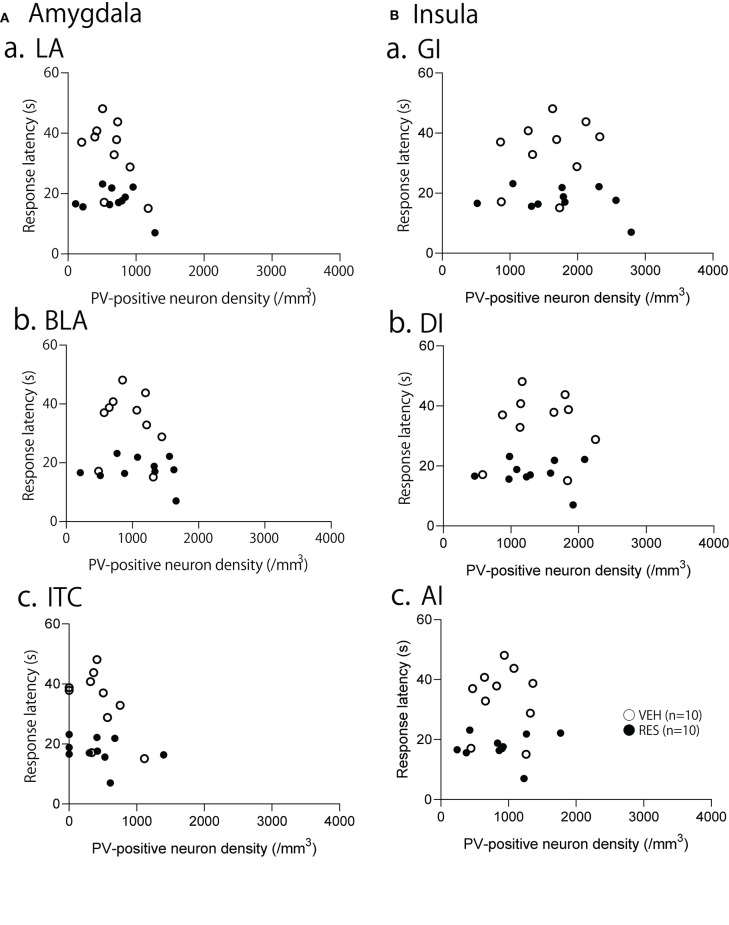
Relationships between response latency in the hot-plate test and PV-positive neuron density in the amygdala **(A)**, and insula cortex **(B)**. **(A)** Relationships between response latency and PV-positive neuron density in the lateral nucleus (LA) (a), basolateral nucleus (BLA) (b), and intercalated cells (ITC) (c) of the amygdala. **(B)** Relationships between response latency and PV-positive neuron density in the granular insula (GI) (a), dysgranular insula (DI) (b), and agranular insula (AI) (c). There were no significant correlations between the response latency and PV-positive neuron density in the all areas (*p* > 0.05).

The relationships between PV-positive neuron density and behavioral manifestation of thermal hyperalgesia (response latency) were analyzed in each brain area. The response latencies in the hot-plate test were significantly negatively correlated with cell density in the S1FL [*r* = −0.680; *F*_(1, 18)_ = 15.50, *p* = 0.001; Figure 4A], S1HL [*r* = −0.645; *F*_(1, 18)_ = 12.80, *p* = 0.002; Figure 4B], and S1L [*r* = −0.677; *F*_(1, 18)_ = 15.19, *p* = 0.001; Figure 4C]. In the other brain regions, no significant relationships were observed. In the mPFC (Figure 5), there was no significant correlation between response latency and PV-positive neuron density in the PrL [*r* = −0.128; *F*_(1, 18)_ = 0.299, *p* = 0.591] (Figure 5A), IL [*r* = −0.289; *F*_(1, 18)_ = 1.638, *p* = 0.217] (Figure 5B), and ACC [*r* = −0.376; *F*_(1, 18)_ = 2.958, *p* = 0.103] (Figure 5C). In the amygdala (Figure 6A), there were no significant correlations between the response latency and PV-positive neuron density in the LA [*r* = −0.333; *F*_(1, 18)_ = 2.245, *p* = 0.151] (Figure 6Aa), BLA [*r* = −0.215; *F*_(1, 18)_ = 0.872, *p* = 0.363] (Figure 6Ab), and ITC [*r* = −0.269; *F*_(1, 18)_ = 1.404, *p* = 0.251] (Figure 6Ac). In the insula cortex (Figure 6B), there were no significant correlations between the response latency and PV-positive neuron density in the GI [*r* = −0.069; *F*_(1, 18)_ = 0.086, *p* = 0.773] (Figure 6Ba), DI [*r* = 0.075; *F*_(1, 18)_ = 0.101, *p* = 0.754] (Figure 6Bb), and AI [*r* = 0.030; *F*_(1, 18)_ = 0.016, *p* = 0.900] (Figure 6Bc).

## Discussion

### Reproduction of the FM Model

A previous study reported that the metabolites of serotonin, dopamine, and noradrenaline in the cerebrospinal fluid (CSF) were lower in patients with FM, suggesting that catecholamine levels may be lower in the brain (47). Consistently, the animal model of FM with repeated reserpine administration replicated human FM symptoms and displayed decreases in catecholamines in the brain and spinal cord (29, 34, 35, 48, 49). The present study also replicated characteristic symptoms of human patients with FM and the animal model of FM reported in previous studies. First, patients with FM often present with eating disorders and/or bodyweight loss (50, 51). Previous animal studies also reported that the FM mouse model displayed the lowest bodyweight 3 days after the first injection (31, 48). After the reserpine administration, access to food was reduced, eating time was extended, and food intake was sharply reduced (52). In the present study, the RES group also showed a decrease in bodyweight 3–5 days after the first injection.

Second, previous studies reported that reserpine-induced changes in pain sensation include mechanical hyperalgesia of the skin and muscles and thermal hyperalgesia. A single dose of reserpine (4 to 5 mg/kg) was found to cause skin and muscle hyperalgesia several hours after injection, and transiently induced thermal hyperalgesia (53, 54). Repeated administration of reserpine resulted in a decrease in the escape threshold for mechanical stimulation of skin and muscle 3 to 14 days after the first injection, while a decrease in escape latency to thermal stimulation was observed 3 to 4 days after the first injection (34, 35, 49). The present results, in which significant thermal hyperalgesia in the hot-plate test was observed 3 days after the first injection, were consistent with those of previous studies.

Third, depression is an important characteristic of human FM, and the same pathophysiological mechanisms may be involved in both depression and changes in pain sensitivity (55, 56). Depression-like symptoms (i.e., immobility in the forced swim test) were not observed 3 days after the first injection of reserpine but were observed 5–14 days after the first injection (29, 34, 35). Consistently, the immobility time tended to increase 4 days after the first injection and was significantly increased 11 days after the first injection in the RES group. These findings indicate that the present study replicated the symptoms of the FM model with repeated reserpine administration.

### Relationship Between PV-Positive Neuron Density and Hyperalgesia

In this study, repeated reserpine administration increased PV-positive neuron density in S1, and there was a negative correlation between PV-positive neuron density and behavioral latency in the hot-plate test. Previous studies reported that optogenetic activation of fast-spike PV-positive neurons controlled pyramidal neuron activity and generated gamma oscillations above 40 Hz (57–59). Consistently, optogenetic activation of PV-positive neurons in the S1 induced gamma oscillations (32). Since gamma oscillations in S1 were correlated with behavioral responses to nociceptive stimuli and gamma oscillations were elevated in S1 in patients with FM (see Introduction), the present results with elevated PV-positive neuron density in S1 and decreases in response latencies in the hot-plate test suggest that gamma oscillations were increased in the RES group. This further suggests that increases in PV-positive neurons in S1 are involved in hyperalgesia.

Optogenetic activation of PV-positive neurons in S1 not only increased behavioral sensitivity to nociceptive stimuli but also markedly increased activity of the rostroventral medulla (RVM), which functions as the descending pain modulatory system (32). It has been demonstrated that the periaqueductal gray (PAG) and RVM in the midbrain regulate nociceptive inputs (60–64). ON and OFF cells are mixed in the RVM. Nociceptive information processing is suppressed by the activity of OFF cells, whereas it is promoted by the activity of ON cells (65–67). In an FM model with reserpine administration, mechanoreceptive C nociceptor responses and activity of dorsal horn microglia in the spinal cord were increased (31), and activated microglia might disinhibit dorsal horn nociceptive neurons (68). Along with the reduction of descending pain-inhibitory catecholaminergic inputs to the spinal cord by reserpine (29, 31), activation of PV-positive neurons in S1 might promote the activity of ON cells in the RVM, most of which might be non-serotonergic (69), to further amplify pain information processing in the dorsal horn.

On the other hand, human fMRI studies reported increased activity in the pre-frontal cortex, anterior cingulate, amygdala, and insula at rest and in response to heat noxious stimuli in patients with FM (70–72). The size of the amygdala changes in patients with FM (73–75). These previous studies suggest that these brain regions might be involved in the pathological processes in FM. However, PV-positive neuron density did not change in these brain regions in the present study. These findings suggest that pathological alterations in PV-positive neurons specifically occur in S1 in an animal model of FM with repeated reserpine administration. However, in the present study, reserpine was administered for only three consecutive days, suggesting that the present results might reflect acute effects. A larger number of reserpine injections would induce changes in other brain regions since sustained changes in catecholamine levels are critical to inducing hyperalgesia (49). Furthermore, in the present study, the animals were sacrificed 3 days after the first injection. Therefore, it is also possible that a longer duration after the first injection might be required to induce changes in PV-positive neurons in other brain regions. Further studies are required to confirm the S1 specificity of PV-positive neuronal changes in FM.

### Possible Pathophysiological Mechanisms of FM by Reserpine

A previous clinical study reported decreases in catecholamine metabolites in the CSF in FM patients, but no alteration of those levels in patients with rheumatoid arthritis, suggesting that alteration of catecholamine metabolites is a cause, but not a consequence, of chronic pain (47). Previous studies reported that catecholamines in the brain suppressed gamma oscillations, whereas their depletion increased gamma oscillations. Dopamine controls gamma oscillations differently depending on its receptor type (76). However, gross depletion of dopamine by pharmacological lesions of dopaminergic terminals in the striatum was found to increase gamma oscillations (77). Furthermore, dopamine reduced gamma oscillation through the **α**1-adrenergic receptor in the primary motor cortex (78). In addition, electrical stimulation of the dorsal raphe nucleus to release serotonin downregulated cortical gamma oscillation (79), while pharmacological stimulation of the locus coeruleus to release noradrenalin reduced gamma oscillation in the dentate gyrus (80). Another line of evidence also indicated an involvement of reserpine in induction of gamma oscillations: reserpine injections increased rapid eye movement (REM) sleep (81), in which gamma oscillations increased compared with non-REM sleep (82). On the other hand, pregabalin, an antagonist of voltage-dependent Ca^2+^ channels (VDCCs), is reported to be effective in treating FM (83). VDCCs are reported to be critical for gamma oscillations in the thalamocortical system (84). All of these findings support the critical role of gamma oscillation in pain information processing in the forebrain of FM. Gamma oscillation is reported to induce synaptic plasticity (85, 86), by which pain sensory circuits might be strengthened in FM. Alteration of PV-positive neurons in the present study may reflect these pathological changes induced by reserpine.

### Limitation

Previous studies reported that non-neuronal cells express PV: ependymal cells in the ventricular wall could express PV in pathological conditions such as brain injury and ventricular stenosis (87, 88). However, PV is a neuronal marker in the brain in intact animals (89, 90). Furthermore, staining distributions of PV-positive cells in the present study were comparable to those of PV-positive neurons observed in the cingulate cortex and reticular thalamic nucleus, as reported previously (91, 92). Although we did not perform double stanning of PV and NeuN, these findings suggest that PV-positive cells were not glial cells but neurons in the present study.

Second, we did not analyze PV-positive neurons in the dorsal horn of the spinal cord, since low frequency oscillations (5–10 Hz) were reported in the dorsal horn (93), compared with high frequency gamma oscillation in the forebrain. However, oscillation frequencies in the dorsal horn could be increased if excitatory inputs to PV-positive neurons in the dorsal horn are increased (94). Reserpine could alter descending projections from the forebrain to the dorsal horn (see above), and consequently increase excitatory inputs to PV-positive neurons, which might lead to activation of PV-positive neurons and induction of gamma oscillations in the dorsal horn. Third, although available information suggest that gamma oscillations may be increased by repeated reserpine injection (see above), there is no direct neurophysiological evidence that repeated reserpine injection induces gamma oscillation in S1 in the present as well-previous studies. Fourth, the present study lacks direct pharmacological evidence indicating that catecholaminergic depletion induces increases in PV-positive neuron density in S1 leading to hyperalgesia. However, indirect evidence supports the present idea: clinical studies reported that serotonin and noradrenaline reuptake inhibitors reduced FM symptoms including hyperalgesia (95–97) while an animal study reported that microinjection of a serotonin reuptake inhibitor into S1 attenuated thermal hyperalgesia (98). To prove or disprove the current idea of a PV-neuronal involvement in FM hyperalgesia, further studies are required to analyze relationships between changes in catecholaminergic levels in the brain and pain sensitivity-related parameters (pain sensitivity, and PV-positive neuron density and gamma oscillations in S1 and the dorsal horn).

## Data Availability Statement

The original contributions presented in the study are included in the article/supplementary material, further inquiries can be directed to the corresponding author/s.

## Ethics Statement

The animal study was reviewed and approved by the Animal Experiments and Ethics Committee at the University of Toyama.

## Author Contributions

HNishij and TT designed the experiment. KM and HNishim performed the experiment. KM, HNishim, and HNishij analyzed the data. KM and HNishij wrote the manuscript. KM, HNishim, JM, TS, TT, TO, and HNishij revised the manuscript. All authors discussed the results, and approved the final manuscript.

## Conflict of Interest

The authors declare that the research was conducted in the absence of any commercial or financial relationships that could be construed as a potential conflict of interest.
